# Design of value-added tax on medicines in 41 European countries: Sabine Vogler

**DOI:** 10.1093/eurpub/ckac131.549

**Published:** 2022-10-25

**Authors:** S Vogler, N Zimmermann

**Affiliations:** WHO Collaborating Centre for Pharmaceutical Pricing and Reimbursement Policies, Austrian National Public Health Institute, Vienna, Austria

## Abstract

**Background:**

Recently, among others due to the COVID-19 pandemic and the war in Ukraine, European countries have been experiencing rising inflation. Value-added taxes (VAT) on essential goods have gained public attention, and abolishing VAT for these goods has been discussed as a measure to prevent poverty and inequities. The study aims to investigate the relevance of value-added tax on medicines in European countries.

**Methods:**

We collated information on medicines-specific and standard VAT rates in 41 countries of the WHO European Region (all 27 European Union Member States, Albania, Armenia, Iceland, Israel, Kazakhstan, Kyrgyzstan, Kosovo, Moldova, North Macedonia, Norway, Serbia, Switzerland, Turkey and United Kingdom). Data were reviewed from literature and were validated by national public authorities.

**Results:**

In three countries (Albania, Kazakhstan and Malta), all medicines are exempt from VAT. 28 of the 38 countries with VAT on medicines impose a single VAT rate on all medicines, and in most of these countries (21 countries) the medicines-related VAT is lower than the standard VAT rate. Ten countries have differentiated VAT rates: In Ireland, Kyrgyzstan, Sweden and the UK defined medicines (e.g. oral medicines, prescription-only medicines) are exempt from VAT, whereas for the other medicines the standard VAT rate applies; six further countries have a lower VAT rate for some medicines (e.g. heparins, blood products, contraceptives or reimbursed medicines in general) compared to the remaining medicines, whose VAT rate equal the standard rate or is lower.

**Conclusions:**

Some European countries apply specific mechanisms (exemptions, reduced rates) regarding the VAT for defined or all medicines. This may act as a protective measure for patients in case of non-reimbursed medicines and help public payers to ensure financial sustainability to funded medicines. Further medicines-specific research is needed to understand the impact of inflation.

**Key messages:**

• Value-added tax is a relevant component of medicine prices.

• Lowering or abolishing the VAT on medicines can be an important policy espeically when patients pay out-of-pocket.

